# PARP inhibitor increases chemosensitivity by upregulating miR-664b-5p in BRCA1-mutated triple-negative breast cancer

**DOI:** 10.1038/srep42319

**Published:** 2017-02-08

**Authors:** Wei Song, Lin Tang, Yumei Xu, Jing Xu, Wenwen Zhang, Hui Xie, Shui Wang, Xiaoxiang Guan

**Affiliations:** 1 Department of Medical Oncology, Jinling Hospital, Southern Medical University, Guangzhou, 510515, China; 2 Department of Medical Oncology, Jinling Hospital, Medical School of Nanjing University, Nanjing, 210002, China; 3 Department of Medical Oncology, Jinling Hospital, Nanjing Medical University, Nanjing, 210002, China; 4 Department of Breast Surgery, The First Affiliated Hospital of Nanjing Medical University, 300 Guangzhou Road, Nanjing, 210029, China

## Abstract

Emerging evidence has shown that adding poly(ADP-ribose) polymerase (PARP) inhibitors to chemotherapy regimens is superior to the control regimens alone in BRCA1-mutated triple-negative breast cancer (TNBC) patients, but their underlying mechanisms have not been fully elucidated. In this study, using miRNA microarray analysis of two BRCA1-mutated TNBC cell lines, we found that miR-664b-5p expression was increased after adding a PARP inhibitor, olaparib, to a carboplatin (CBP) plus gemcitabine (GEM) therapy regimen. Functional assays showed miR-664b-5p overexpression inhibited proliferation, migration and invasion in BRCA1-mutated TNBC cells. CCNE2 was identified as a novel functional target of miR-664b-5p, and CCNE2 knockdown revealed effects similar to those observed with miR-664b-5p overexpression. Both CCNE2 knockdown and miR-664b-5p overexpression significantly increased the chemosensitivity of BRCA1-mutated TNBC cells. In addition, *in vivo* studies indicated that miR-664b-5p inhibited tumour growth compared with the control in tumour xenograft models, and we also found that CCNE2 expression was inversely correlated with miR-664b-5p expression in 90 TNBC patient samples. In conclusion, miR-664b-5p functions as a tumour suppressor and has an important role in the regulation of PARP inhibitors to increase chemosensitivity by targeting CCNE2. This may be one of the possible mechanisms by which PARP inhibitors increase chemosensitivity in BRCA1-mutated TNBC.

TNBC is a special subtype of breast cancer that lacks oestrogen receptor (ER), progesterone receptor (PR), and human epidermal growth factor receptor type 2 (HER2) gene expression, all of which are molecular targets of therapeutic agents^1^. Patients with TNBC typically have a relatively poorer outcome compared with those with other breast cancer subtypes due to the distinctly aggressive clinical behaviour and the lack of recognized molecular targets for therapy^2^,^3^. Therefore, chemotherapy is the primary established treatment option for patients with TNBC^4^. In recent years, a high level of heterogeneity in TNBCs has been revealed, such as germline BRCA1/2 mutations^2^,^5^,^6^,^7^. Many studies have focused on identifying potentially actionable molecular features for treatment of TNBC^8^,^9^,^10^,^11^.

Unfortunately, previous trials on monotherapy with PARP inhibitors in TNBC patients have not been as successful as anticipated^12^. Thus, further trials should primarily concentrate on the selection of the patient population and appropriate combination regimens for optimal disease control. Many clinical trials on platinum-based chemotherapy have confirmed that platinum compounds have a relevant role in the treatment of TNBC patients, especially those harbouring BRCA1/2 mutations^4^,^13^,^14^,^15^. For these reasons, many studies on platinum-based chemotherapy combined with a PARP inhibitor are being performed^16^,^17^. A phase 3 study evaluating the safety and efficacy of the addition of veliparib with carboplatin versus the addition of carboplatin to standard neoadjuvant chemotherapy versus standard neoadjuvant chemotherapy in early-stage TNBC patients with a documented BRCA germline mutation is ongoing (ClinicalTrials.gov NCT02032277). Thus far, the results indicate that combination regimens that include a PARP inhibiter are preferable to platinum-based chemotherapy in BRCA1-mutated TNBC. In addition, it is interesting to note that the addition of a PARP inhibiter to cyclophosphamide did not improve the response rate over cyclophosphamide alone^18^. However, mechanisms underlying the combination of chemotherapy and PARP inhibition are not fully understood.

MicroRNAs (miRNAs) comprise approximately 22 nucleotides and are a class of non-coding RNAs that down-regulate target gene expression post-transcriptionally by binding to the 3′ untranslated region (3′UTR) of mRNA. They function in numerous important pathophysiological processes, such as regulating cell proliferation, differentiation, migration, and apoptosis, and participate in the regulation of chemotherapy resistance and sensitivity in many human cancers, including breast cancer^19^,^20^,^21^,^22^,^23^. Dysregulation of miRNAs is reported to be involved in the chemotherapy sensitivity of breast cancer. Yang *et al*. have reported that upregulation of miR-195 increases the sensitivity of breast cancer cells to adriamycin treatment through inhibition of Raf-1^24^. Moreover, drugs can exert therapeutic effects through modulation of the expression of miRNAs. For example, research indicates that trastuzumab suppresses breast cancer cell growth by upregulating miR-26a and miR-30b^25^.

In the present study, we screened the differentially expressed miRNAs in two BRCA1-mutated TNBC cell lines, which were exposed to CBP plus GEM with or without the PARP inhibitor olaparib. Of these screened miRNAs, miR-664b-5p was selected for further study. Furthermore, we investigated the functions and mechanisms of miR-664b-5p and its target gene CCNE2 *in vitro* and *in vivo*. We found that miR-664b-5p functioned as a tumour suppressor and had an important role in the sensitivity to chemotherapy by targeting CCNE2. This may be one of the possible mechanisms that chemotherapy plus a PARP inhibiter is significantly superior to chemotherapy alone, which may be a potential strategy for future therapies for BRCA1-mutated TNBC.

## Results

### Adding a PARP inhibitor to CBP plus GEM upregulates miR-664b-5p expression

**Figure 1:**
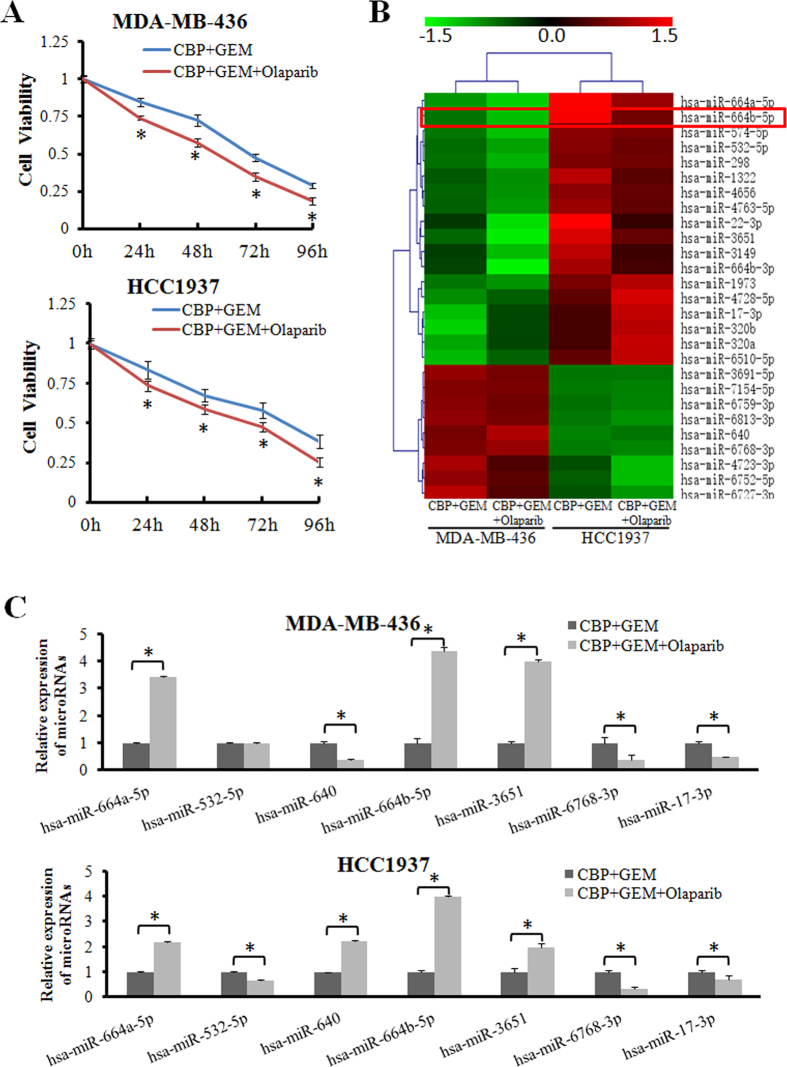
Adding PARP inhibitor to CBP plus GEM upregulates miR-664b-5p expression. (**A**) The BRCA1-mutated TNBC cell lines MDA-MB-436 and HCC1937 were treated with CBP (10 μM) plus GEM (100 nM) combined with or without olaparib (10 μM) for the indicated times, and then, the cell viability was determined with an MTT assay. The results show data from six independent experiments expressed as the mean ± SD. p < 0.05. (**B**) A miRNA microarray was performed to detect differentially expressed miRNAs of MDA-MB-436 and HCC1937 cells treated with CBP plus GEM combined with or without olaparib for 72 h. The green in the legend represents down-regulation, and the red represents upregulation (>1.5-fold change in expression, p < 0.05). (**C**) Validation by qRT–PCR of candidate miRNAs that showed significant differential expression in the miRNA microarray. The error bars represent the mean of three separate determinations ± the standard deviation (SD). *p < 0.05.

To investigate the effects of chemotherapy with the PARP inhibitor olaparib or chemotherapy alone on cell proliferation, an MTT assay was performed. We confirmed that CBP plus GEM combined with the PARP inhibitor olaparib significantly suppressed the proliferation of two BRCA1-mutated TNBC cell lines, MDA-MB-436 and HCC1937, compared with CBP plus GEM (p < 0.05) (Fig. 1A), but this action of proliferation inhibition was not observed in the non-BRCA1-mutated TNBC cell line, MDA-MB-231 (Supplementary Fig. S1). Then, we performed a miRNA microarray in MDA-MB-436 and HCC1937 cells after CBP plus GEM with or without olaparib treatment (Fig. 1B). According to the microarray results, the top seven most differentially expressed miRNAs (miR-664a-5p, miR-532-5p, miR-640, miR-664b-5p, miR-3651, miR-6768-3p, miR-17-3p), which were consistently changed in both cell lines, were further validated by quantitative real-time polymerase chain reaction (qRT-PCR). As shown in Fig. 1C, the expression levels of five (miR-664a-5p, miR-664b-5p, miR-3651, miR-6768-3p, miR-17-3p) out of the seven miRNAs were consistent with the microarray results, among which the difference in miR-664b-5p expression was the most remarkable. There was no difference of the expression of miR-664b-5p observed in MDA-MB-231 (Supplementary Fig. S2). Therefore, miR-664b-5p was selected for further study.

### miR-664b-5p overexpression suppresses cell growth, migration and invasion

**Figure 2:**
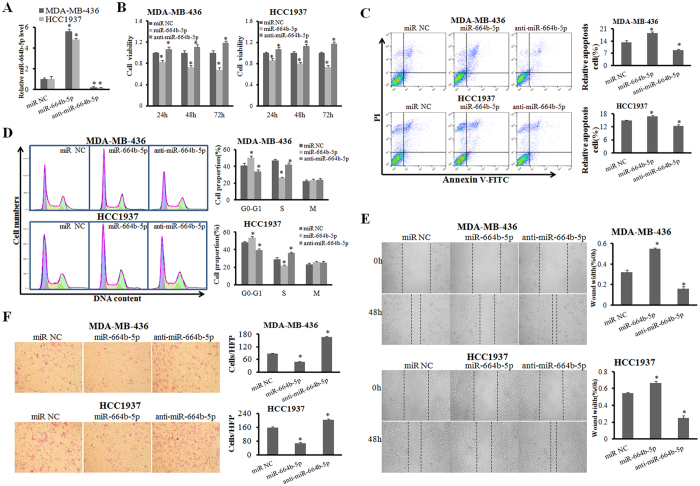
miR-664b-5p overexpression suppresses cell growth, migration and invasion. (**A**) The efficiency of miR-664b-5p overexpression and inhibition in BRCA1-mutated TNBC cell lines was measured with qRT-PCR. (**B**) The influence of miR-664b-5p on the cell growth of BRCA1-mutated TNBC cells was measured with an MTT assay. (**C**) The representative images of cell apoptosis analysed by flow cytometry using Annexin V and PI staining. The apoptotic rate in MDA-MB-436 and HCC1937 cells transfected with miR-NC, miR-664b-5p, or anti-miR-664b-5p. (**D**) Representative images of the cell cycle analysis by flow cytometry using Annexin V and PI staining. The cell-cycle phase distribution of MDA-MB-436 and HCC1937 cells transfected with miR-NC, miR-664b-5p, or anti-miR-664b-5p. (**E**) Wound-healing assays in MDA-MB-436 and HCC1937 cells were performed after transduction with miR-NC, miR-664b-5p, or anti-miR-664b-5p. (**F**) Representative results of the Transwell assays showing the effect of miR-664b-5p expression on the invasion ability in MDA-MB-436 and HCC1937 cells. *p < 0.05.

To examine the role of miR-664b-5p in cell growth, MDA-MB-436 and HCC1937 cells were treated separately with miR-NC, miR-664b-5p mimics and anti-miR-664b-5p. Transfection of miR-664b-5p mimics significantly increased miR-664b-5p levels and the anti-miR-664b-5p sharply decreased the miR-664b-5p levels (p < 0.05) (Fig. 2A). MTT assays showed that miR-664b-5p overexpression distinctly inhibited the cell growth compared with the control *in vitro* (p < 0.05) (Fig. 2B). In contrast, miR-664b-5p suppression significantly promoted cell growth. Next, the effect of miR-664b-5p on the cell cycle was analysed. Following the forced expression of miR-664b-5p, the quantity of cells in the G1 phase increased significantly and the percentage of cells in S phase decreased in both MDA-MB-436 and HCC1937 cells (p < 0.05) (Fig. 2C). This illustrated that G1-to-S-phase transition was inhibited by miR-664b-5p overexpression. In contrast, miR-664b-5p suppression led to a reverse cell cycle pattern. The number of apoptotic cells after transfection was then assessed. The ratio of apoptotic cells was increased following overexpression of miR-664b-5p compared with the control in both MDA-MB-436 and HCC1937 cells (p < 0.05). miR-664b-5p suppression induced a decrease in cell apoptosis (Fig. 2D). We next investigated whether miR-664b-5p had an effect on the motility and invasiveness properties of the two BRCA1-mutated TNBC cell lines. As shown in Fig. 2E and F, miR-664b-5p overexpression significantly decreased the migration ability of MDA-MB-436 and HCC1937 cells and weakened the invasive potential of these cells (p < 0.05). The inhibition of miR-664b-5p by anti- miR-664b-5p promoted cell migration and invasion in both cell lines. Taken together, our results showed that miR-664b-5p overexpression suppressed cell growth, induced cell cycle arrest and apoptosis, and inhibited cell migration and invasion.

### CCNE2 is a direct downstream target of miR-664b-5p

**Figure 3:**
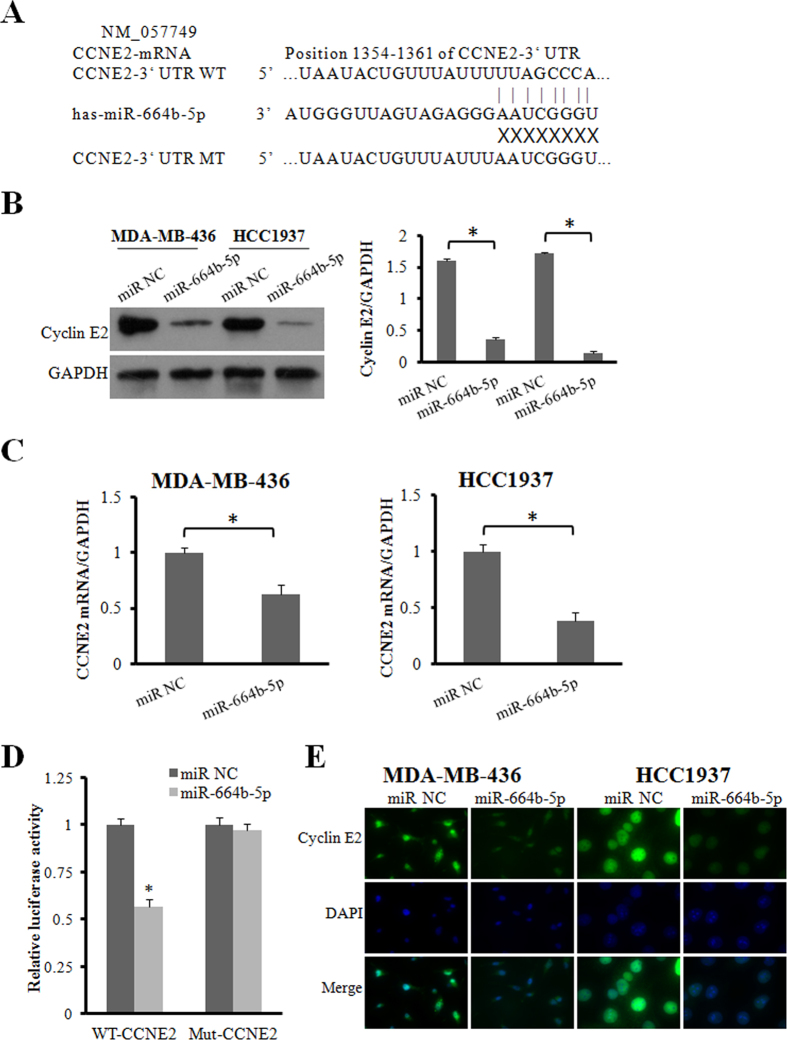
CCNE2 is a direct downstream target of miR-664b-5p. (**A**) The nucleotide sequences of miR-664b-5p and the complementary sequence in CCNE2 mRNA revealed a potential binding site. (**B**) Western blot analysis of cyclin E2 protein in miR-664b-5p overexpressing cells. GAPDH expression was used as the loading control. (**C**) qRT-PCR detection of CCNE2 mRNA expression in MDA-MB-436 and HCC1937 cells transfected with miR-664b-5p or miR-NC. (**D**) Relative luciferase activity was analysed after wild-type or mutant 3′-UTR reporter plasmids were cotransfected with miR-664b-5p or miR-NC in HCC1937 cells. (**E**) Fluorescence microscopy analysis of the expression of cyclin E2 by immunofluorescence. The green signal represents the staining of the cyclin E2 protein, and the blue signal represents the nuclear DNA staining by DAPI. *p < 0.05.

It is well recognized that miRNAs function by regulating the expression of target genes. To investigate the molecular mechanisms underlying the effect of miR-664b-5p on the two BRCA1-mutated TNBC cells, two miRNA databases (TargetScan and miRDB) were used to predict the miR-664b-5p binding sites in human mRNA transcripts. We found that CCNE2 was predicted by both predictive tools (Fig. 3A). Cyclin E2, an E-type cyclin family member, is encoded by the CCNE2 gene and participates in proliferation, invasion and metastasis by activating downstream signalling pathways. Therefore, the next step was to validate whether miR-664b-5p functioned as a tumour suppressor by regulating CCNE2. We found that the protein expression and mRNA levels of CCNE2 were down-regulated in both MDA-MB-436 and HCC1937 cells when miR-664b-5p was overexpressed compared with the control cells (p < 0.05). The expression levels of CCNE2 mRNA and protein were increased in MDA-MB-436 cells transfected with miR-664b-5p inhibitors (p < 0.05) and were also observed to increase in HCC1937 cells (Fig. 3B,C,E). Taken together, we verified that the down-regulation of CCNE2 was caused by overexpression of miR-664b-5p. To confirm whether miR-664b-5p regulated CCNE2 expression by directly binding to the CCNE2 3′UTR, the wild-type and mutant 3′UTR region of CCNE2 was cloned downstream of the firefly luciferase gene in pmirGLO reporters. Luciferase assays showed that a significant (41%) decrease in luciferase activity was observed when cells were co-transfected with miR-664b-5p mimics compared to the miR-NC in the wild-type 3′UTR group (p < 0.05), while in the mutant 3′UTR and empty control groups, this luciferase activity was abrogated when the HCC1937 cells were co-transfected with miR-664b-5p mimics (Fig. 3D). These results suggest that the CCNE2 gene is a functional target of miR-664b-5p and that miR-664b-5p directly targeted putative CCNE2 3′UTR regions.

### CCNE2 knockdown revealed effects similar to those observed with ectopic miR-664b-5p expression

**Figure 4:**
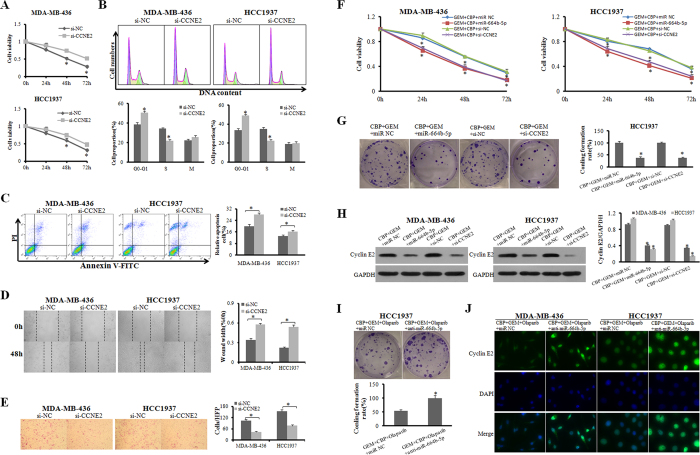
CCNE2 knockdown revealed similar effects as that of ectopic miR-664b-5p expression. (**A**) The influence of CCNE2 on the cell growth of BRCA1-mutated TNBC cells was measured with an MTT assay. (**B**,**C**) The representative images of cell apoptosis and cell cycle were analysed by flow cytometry in MDA-MB-436 and HCC1937 cells transfected with si-CCNE2 or si-NC. (**D**,**E**) Wound-healing and Transwell assays in MDA-MB-436 and HCC1937 cells were performed after transduction with si-CCNE2 or si-NC. (**F**) The influence of adding miR-664b-5p overexpression or CCNE2 interference to CBP plus GEM treatment on the cell growth of BRCA1-mutated TNBC cells was measured with an MTT assay. (**G**) Colony formation of HCC1937 cells transfected with miR-664b-5p mimics or si-CCNE2 after exposure to CBP plus GEM for 72 h. (**H**) Western blot analysis of cyclin E2 protein in miR-664b-5p-overexpressing cells. GAPDH expression was used as the loading control. (**I**) Colony formation of HCC1937 cells transfected with miR-664b-5p inhibitors or miR-NC after exposure to CBP plus GEM combined with olaparib for 72 h. (**J**) Fluorescence microscopy analysis of the expression of cyclin E2 by IF. The green signal represents the staining of cyclin E2 protein, and the blue signal represents the nuclear DNA staining by DAPI. *p < 0.05.

To explore whether CCNE2 down-regulation by miR-664b-5p was responsible for the tumour suppression of this miRNA, we performed gain-of-function and loss-of-function analyses. MDA-MB-436 and HCC1937 cells were transfected with CCNE2 siRNAs (si-CCNE2) or a negative control siRNA (si-NC), and western blotting demonstrated that CCNE2 expression was inhibited by its specific siRNAs. CCNE2 knockdown suppressed cell growth, migration and invasion (Fig. 4A,B and C), blocked G1-to-S-phase transition, and slightly promoted apoptosis compared with the control (p < 0.05) (Fig. 4D and E). Next, to assess the functions of miR-664b-5p and CCNE2 in regulating sensitivity to chemotherapy, cell proliferation and colony formation ability were detected after treatment with CBP plus GEM and then transfection with miR-NC, miR-664b-5p mimics or si-CCNE2. The results of MTT assays indicated that cell viability was strongly inhibited by CBP plus GEM in MDA-MB-436 and HCC1937 cells transfected with miR-664b-5p mimics or si-CCNE2 (p < 0.05) (Fig. 4F). The survival fractions of HCC1937 cells transfected with miR-664b-5p mimics or si-CCNE2 were lower than those of control cells (p < 0.05) (Fig. 4G). The cyclin E2 protein levels were markedly reduced in CBP plus GEM-treated HCC1937 cells transfected with miR-664b-5p mimics or si-CCNE2, compared with CBP plus GEM-treated miR-NC-transfected cells (Fig. 4H). Anti-miR-664b-5p weakened the growth inhibition caused by CBP plus GEM combined with the PARP inhibitor olaparib in BRCA1-mutated TNBC cells. Moreover, we explored the cyclin E2 expression after treatments by immunofluorescence (IF) (Fig. 4I and J). The results showed that the chemotherapy treatment plus PARP inhibition reduced the expression of cyclin E2. These data demonstrated that both elevated miR-664b-5p expression and knockdown of CCNE2 decreased cell viability and suppressed cell proliferation, resulting in increased sensitivity of BRCA1-mutated TNBC cells to CBP plus GEM. miR-664b-5p and CCNE2 are involved in the mechanisms of the sensitivity to chemotherapy plus PARP inhibition.

### Effect of miR-664b-5p on tumour growth *in vivo*

**Figure 5:**
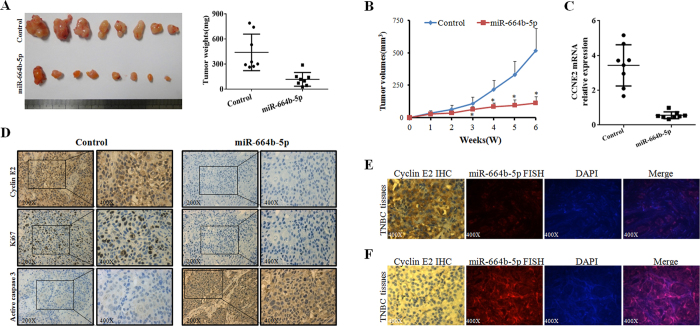
Effect of miR-664b-5p on tumour growth *in vivo*. (**A**) The gross morphology of tumours and the final xenograft tumour weights measured on day 42 after injection of tumour cells. (**B**) Effect of miR-664b-5p on tumour growth *in vivo*. Growth curves of HCC1937 subcutaneous xenograft tumours treated with miR-664b-5p or miR-NC. Tumour volumes were calculated as (long axis × short axis^2^)/2; n = 8 per group. (**C**) qRT-PCR detection of CCNE2 mRNA expression in two groups of tumour xenografts. (**D**) Representative images of the expression of cyclin E2, Ki67 and active caspase-3 in two groups of tumour xenografts determined by IHC. (**E**,**F**) Representative images of cyclin E2 high-expression (IHC) and miR-664b-5p low-expression (FISH) (E) or cyclin E2 low-expression and miR-664b-5p high-expression (**F**) in TNBC patient specimens. *p < 0.05.

To further identify the effect of miR-664b-5p *in vivo*, we performed a tumour xenograft study. HCC1937 cells were treated with a lentiviral miR-664b-5p vector or empty vector. In contrast, in mice treated with miR-664b-5p overexpressed cells, tumour growth was significantly delayed, with a 78% (p < 0.01) reduction in tumour volume at day 42 compared with the control (miR-664b-5p group: mean tumour volume = 113.40 mm^3^, 95% CI = 54.78 to 172.02 mm^3^; control group: mean tumour volume = 519.37 mm^3^, 95% CI = 290.76 to 747.98 mm^3^) (Fig. 5A). Moreover, tumour weights on day 42 after tumour cell injection were significantly lower than the control, with a 73% (p < 0.01) reduction in weight (miR-664b-5p group: mean tumour weight = 117 mg, 95% CI = 49.16 to 185.98 mg; control group: mean tumour weight = 439.72 mg, 95% CI = 256.90 to 622.54 mg) (Fig. 5B). Furthermore, the results of PCR and immunohistochemistry (IHC) displayed lower CCNE2 mRNA and protein levels in miR-664b-5p–overexpressing tumour xenografts. miR-664b-5p-overexpressing tumours showed lower Ki67 and higher active caspase-3 staining by IHC, which indicated that miR-664b-5p–overexpressing tumours had lower proliferative and higher apoptotic potential (Fig. 5C and D).

To further analyse the correlation between miR-664b-5p and CCNE2 expression, we examined miR-664b-5p expression by fluorescence *in situ* hybridization (FISH) and cyclin E2 protein expression by IHC in 90 TNBC tissue specimens (Fig. 5E and F). Of the 90 TNBC tissues, 54 cases (60.0%) and 36 cases (40.0%) expressed cyclin E2 at high and low levels, respectively, while 40 cases (44.4%) and 50 cases (55.6%) expressed miR-664b-5p at high and low levels, respectively (Table 1). There was a significant negative correlation between the expression of cyclin E2 and miR-664b-5p (r = −0.502, p < 0.001) (Table 2). Therefore, these data suggested that miR-664b-5p played a positive role in inhibiting tumour growth *in vivo*.

## Discussion

An increasing body of evidence has demonstrated that chemotherapy combined with PARP inhibition improves the curative effects compared to chemotherapy alone in BRCA1-mutated cancers, including TNBC^16^,^26^. In BRCA-mutant models, CBP plus PARP inhibition revealed a modest survival advantage versus CBP alone. We also observed a greater growth suppression effect when the BRCA1-mutated TNBC cells, MDA-NB-436 and HCC1937, were exposed to CBP plus GEM combined with olaparib compared to the CBP plus GEM exposure. miRNAs have emerged as potent and plausible regulators that are involved in complex physiologic and pathologic processes. Dysfunction or dysregulation of miRNAs is reported to be related to the resistance and sensitivity to chemotherapy^19^,^22^, for instance, microRNA-224 promotes the sensitivity of osteosarcoma cells to cisplatin by targeting Rac1^27^, and microRNA-205 increases the sensitivity of breast cancer to docetaxel^19^.

In the present study, we aimed to investigate what role miRNAs played in the difference between the effectiveness of chemotherapy combined with PARP inhibition and chemotherapy alone in BRCA1-mutated TNBC. Using a miRNA microarray and RT-PCR, we detected miR-664b-5p as a likely important regulating factor. There are few research studies on the functions of miR-664b-5p in cancer. We found that miR-664b-5p overexpression increased cell proliferation and inhibited migration and invasion in BRCA1-mutated TNBC cells.

Cyclin E2 is the second member of the E-type cyclin family. Aside from its specific function as a regulator of G1-to-S-phase transition, cyclin E2 also plays a direct role in the initiation of DNA replication, the control of genomic stability, and the centrosome cycle^28^. CCNE2 overexpression has been found in various types of cancer, such as lung cancer, gastric cancer, pancreatic cancer, head and neck squamous cell carcinomas and breast cancer^29^,^30^. Moreover, activation of CCNE2 can increase cancer cell migration, adhesion, invasion, proliferation and metastasis^31^. Previous studies have reported that cyclin E2 participated in the regulation of invasion and metastasis by activating downstream signalling pathways^31^,^32^,^33^. We identified CCNE2 as a direct target gene of miR-664b-5p in BRCA1-mutated TNBC. Furthermore, miR-664b-5p overexpression or CCNE2 interference increased the sensitivity of BRCA1-mutated TNBC cells to CBP plus GEM. Anti-miR-664b-5p weakened the effect of chemotherapy combined with PARP inhibition on the suppression of cell growth. A tumour xenograft study verified that miR-664b-5p plays a positive role in inhibiting tumour growth *in vivo*. The correlation analysis showed a significant negative correlation between the presence of CCNE2 expression and miR-664b-5p.

In addition, although we observed that miR-664b-5p effectively inhibited tumour growth in the mouse xenograft model by subcutaneous injection, it was better to use a tail vein injection method, in view of the character of TNBC invasion, to evaluate miR-664b-5p effects *in vivo*. In addition, more studies are needed, for example, studies of the concrete mechanism by which miR-664b-5p expression was increased after the chemotherapy treatment combined with PARP inhibition and studies to determine the correlation between miR-664b-5p and the clinical features, such as progression free survival (PFS), etc. In conclusion, we found for the first time, to the best of our knowledge, that miR-664b-5p functions as a tumour suppressor and has an important role in chemotherapy sensitivity by targeting CCNE2 in BRCA1-mutated TNBC. This may be one of the possible mechanisms that allows chemotherapy plus PARP inhibition to be significantly superior to chemotherapy alone, which may be a potential strategy for future therapies in BRCA1-mutated TNBC patients.

## Materials and Methods

### Clinical specimens and Ethics statement

In total, 90 TNBC tissue samples were collected from patients who underwent tumour resection in The First Affiliated Hospital of Wenzhou Medical University from April 2005 to March 2014. The study protocol was approved by the Ethics Committee of Jinling Hospital. Histological parameters were determined in accordance with the criteria of the World Health Organization. All patients gave their informed consent prior to inclusion in the study. Specimens and all experimental procedures were handled and performed in accordance with the approved guidelines.

### Cell lines, Animals and Reagents

The BRCA1-mutated human TNBC cell lines MDA-MB-436 and HCC1937 were obtained from the American Type Culture Collection (ATCC, Manassas, VA, USA). Cells were cultured in RPMI-1640 medium (Gibco, USA) supplemented with 10% foetal bovine serum (FBS) (HyClone, USA) and then incubated in a humidified atmosphere with 5% CO_2_ at 37 °C. Gemcitabine, carboplatin and olaparib were purchased from Selleck Chemicals (Houston, TX, USA). BALB/c athymic nude mice (female, 6 weeks old) were purchased from the Department of Comparative Medicine, Jinling Hospital (Nanjing, China) and maintained in a pathogen-free facility. Animal studies were performed in accordance with institutional guidelines.

### miRNA microarray

Total RNA was isolated from MDA-MB-436 and HCC1937 cells treated with GEM plus CBP with or without olaparib for 72 h using TRIzol (Invitrogen, USA) according to the manufacturer’s instructions. Small RNA was extracted by using a mirVana kit (Ambion, Austin, USA) and subsequently labelled with Cy3 and Cy5 fluorescent dyes, which were applied for hybridization to dual-channel microarrays on each chip consisting of 2576 probes to detect 1321 human miRNAs. The raw data were normalized and adjusted using GenePix Pro 4.0 software, and obvious outliers were removed. The miRNA expression in the experimental group (treated with CBP plus GEM with olaparib) was compared with the expression in the corresponding control group (treated with CBP plus GEM), and the fold-change was calculated. Student’s t-test analyses were performed to compare the experimental and control groups in the same cell line, and miRNA with p values of <0.05 were chosen for cluster analysis using a hierarchical method.

### RNA extraction and qRT-PCR

Total RNA was extracted from cultured cells and tumour xenografts using TRIzol. For qRT-PCR analysis of CCNE2 mRNA expression, the cDNA was synthesized using a PrimeScriptTM RT Master Mix (Perfect Real Time) Kit (RR036A, Takara, China), followed by PCR using Power SYBR Green PCR Master Mix (Life Technology, USA). GAPDH was used as an internal control. The primer sequences were as follows: CCNE2-forward, 5′-GCCGAGCGGTAGCTGGTC-3′ and reverse, 5′-GGGCTGCTGCTTAGCTTGTAAA-3′; GAPDH-forward, 5′-AAATCAAGTGGGGCGATGCTG-3′ and reverse, 5′-GCAGAGATGATGACCCTTTTG-3′. For analysis of miR-664b-5p expression, mature miRNA from tissues and cells was extracted by using a mirVana kit (Ambion, Carlsbad, MA), and the expression levels of miR-664b-5p were detected using Power SYBR Green PCR Master Mix; U6 small nuclear RNA was used as an internal control. The relative expression of CCNE2 and miR-664b-5p was determined using the 2^−ΔΔCt^ method.

### Cell viability and Colony formation assays, Cell cycle and Apoptosis analyses

Cell viability was measured with an MTT assay. Briefly, 1 × 10^3^ cells were seeded into a 96-well plate with 6 repeats for each condition. After 12 h, the cells were treated with GEM plus CBP with or without olaparib. Then, cells were cultured for 24, 48, and 72 h after treatments. MTT solution (10 μl, 5 mg/ml) (Sigma, USA) was added into each well and incubated at 37 °C. After 4 h, the supernatants were removed and 100 μl of DMSO (Sigma, USA) was added to each well. The absorbance value (OD) of each well was measured at 490 nm.

The HCC1937 cells transfected and/or treated with medication were diluted and replaced in six-well plates at 500 cells per well. After incubating for 14 days, cells were fixed with 100% methanol and stained with 0.5% crystal violet. Colonies containing more than 50 cells were counted as survivors. Survival curve parameters were determined by a linear-quadratic equation. The experiments were performed at least three times.

Cells were incubated with culture medium for 48 h after transfection with miR-664b-5p mimics, inhibitors or negative control in 6-well plates at 2 × 10^5^ per well. For apoptosis analysis, the cells were collected, washed with PBS, resuspended in 100 μl of 1 × binding buffer and stained with 5 μl of Annexin V and 5 μl of PI (Becton-Dickinson) for 15 min at room temperature in the dark. For cell cycle analysis, cells were washed with ice-cold PBS and fixed with 70% ethanol overnight at −20 °C. Fixed cells were rehydrated in PBS for 10 min and subjected to PI/RNase staining. A flow cytometer (Becton-Dickinson) was utilized to evaluate the apoptotic levels and cell cycle in each sample following the manufacturer’s instructions. Then, the data were analysed using FlowJo, V10 software.

### Transfection

The MDA-MB-436 and HCC1937 cells were transfected with miR-664b-5p mimics (5′-UGGGCUAAGGGAGAUGAUUGGGUA-3′), negative control oligonucleotide (miR-NC, 5′-UCCUCCGAACGUGUCACGUTT-3′), 2′-O-methyl modified miR-664b-5p inhibitor (anti-miR-664b-5p, 5′-UACCCAAUCAUCUCCCUUAGCCCA-3′), CCNE2 siRNA pool (3 siRNAs mixed in an equimolar ratio), (si-CCNE2, 1-5′-AGACGAAGUAGCCGUUUAC-3′, 2-5′-GGAUGGAACUCAUUAUAUU-3′, and 3-5′- GUUGGCCACCUGUAUUAUC-3′) and NC siRNA (si-NC, 5′-UUCUCCGAACGUGUCACGU-3′) using Lipofectamine 2000 (Invitrogen, USA), according to the manufacturer’s guidance. HCC1937 cells were infected with the resultant recombinant lentivirus plasmid LV3-hsa-miR-664b-5p or LV3NC in the presence of 6 μg/ml polybrene (Sigma-Aldrich). All of oligonucleotides and lentivirus plasmids were purchased from Genepharma Company (China).

### Wound-healing, Migration and Invasion assays

Cells were seeded in six-well plates and incubated to generate confluent cultures. Wounds were scratched in the cell monolayer using a 200 μl sterile pipette tip. The cells were rinsed with PBS. The migration of the cells at the edge of the scratch was photographed at time 0 and at 48 h.

The invasion ability of MDA-MB-436 and HCC1937 cells was determined using 24-well Transwell chambers (Costar, USA) coated with Matrigel (BD Pharmingen, San Jose, CA). After transfection, approximately 1 × 10^5^ cells/200 μl were plated in medium without serum in the top chamber of each Transwell. Then, 800 μl of medium supplemented with 10% foetal bovine serum was injected into the lower chamber. After 24 h of incubation, the inserts were fixed with 100% methanol, subsequently stained with crystal violet and photographed under a microscope.

### Luciferase assay

HCC1937 cells were cultured in 24-well plates and co-transfected with 20 ng of the pmirGLO-CCNE2-3′-UTR vector and 5 pmol of either the miR-664b-5p mimics or the control mimics. After 48 h of incubation, firefly and Renilla luciferase activities of the cell lysates were measured using a Dual-Luciferase Reporter Assay System (Promega).

### Western blot and Immunofluorescence

Cell lysates were resolved by SDS-PAGE electrophoresis (30 μg/sample) and electro-transferred onto polyvinylidene fluoride (PVDF) membranes. After incubation in blocking buffer, the membranes were probed overnight at 4 °C with the primary antibodies: GAPDH, 1: 5000 (CST); cyclin E2 [EP454Y] (ab40890), 1: 1000 (Abcam). The subsequent steps were performed as previously described^34^.

Cells were fixed with 3% paraformaldehyde for 10 min, permeabilized with 0.1% SDS solution in PBS for 10 min, and then blocked with FCS 10% solution in PBS for 20 min. Fixed cells were stained with an anti-cyclin E2 (1: 300) and then with a secondary antibody coupled to Dylight 488 for 30 min. Cell nuclei were stained with DAPI-Fluoromount-G for 15 min. The expression was then defined using the following parameters: -, no immunofluorescence; ±, weak immunofluorescence; +, moderate immunofluorescence; ++, strong immunofluorescence; and; +++, very strong immunofluorescence. The samples with a score of ++ or +++ were defined as high expressing; all other samples were considered low expressing.

### Immunohistochemistry

Following deparaffinization, sections were rehydrated and subjected to antigen retrieval using citrate buffer (BioGenex, USA). The slides were incubated with cyclin E2 primary antibody (1: 200) at 4 °C overnight. The following steps were performed as previously described^35^. The percentages of positive cells and staining intensities were scored as previously described^34^. IHC scoring was performed without prior knowledge of the clinical response.

### Fluorescence *in situ* hybridization

Human TNBC tissue arrays were dewaxed in xylene, rehydrated through an ethanol series (100%, 95%, 90%, 80%, 70%, 50%, 30%) and postfixed in 4% paraformaldehyde for 30 min. Then, the arrays were digested with proteinase K (10 μg/ml, Roche) for 5 min, refixed with 4% paraformaldehyde, and washed in Tris/glycine buffer. After that, the arrays were hybridized overnight at 60 °C within coverslip chambers in hybridization buffer, containing 1 ng/μl of fluorescein-labelled LNA probes. After hybridization, the arrays were rinsed once with 5 × SSC and once with 0.5 × SSC at 25 °C for 1 h. The fluorescence was scored as previously described.

### Tumour xenograft study

All animal experiments were conducted in accordance with the Guide for the Care and Use of Laboratory Animals, and all experimental protocols were approved by the Animal Ethics Committee. To form tumour xenografts in female six-week-old BALB/c nude mice, empty or lentiviral miR-664b-5p vector transfected-HCC1937 cells (1 × 10^7^) were injected subcutaneously in the back under aseptic conditions. Tumour size was measured using callipers every week, and tumour volume was calculated according to the following equation: (long axis × short axis^2^)/2. The xenograft tumours were removed on the sixth week after the injection and weighed after dissection.

### Statistical analysis

Data from at least three independent experiments are presented as the means ± standard error of the mean. Differences between groups were calculated by Student’s t-test or one-way analysis of variance using an SPSS 19.0 software package (SPSS Inc.). The association between CCNE2 and miR-664b-5p expression in TNBC tissues was explored by chi-square test. p < 0.05 was considered to indicate a statistically significant difference.

## Additional Information

**How to cite this article:** Song, W. *et al*. PARP inhibitor increases chemosensitivity by upregulating miR-664b-5p in BRCA1-mutated triple-negative breast cancer. *Sci. Rep.* **7**, 42319; doi: 10.1038/srep42319 (2017).

**Publisher's note:** Springer Nature remains neutral with regard to jurisdictional claims in published maps and institutional affiliations.

## Supplementary Material

Supplementary Figures

## Figures and Tables

**Table 1:** Relationship between expression of cyclin E2, miR-664b-5p and clinicopathologic characteristics of TNBC patients.

Variables	Cyclin E2				*P-v*alue	miR-664b-5p				*P-*value
	high(n = 54)		low(n = 36)			high(n = 40)		low(n = 50)		
	No.	%.	No.	%		No.	%	No.	%	
**Age(years)**					0.667					0.671
≦48	26	48.1	19	52.8		21	52.5	24	48.0	
>48	28	51.9	17	47.2		19	47.5	26	52.0	
**Tumour size(cm)**	13	24.1	18	50.0	0.022*	19	47.5	12	24.0	0.029*
≤2	38	70.4	18	50.0		21	52.5	35	70.0	
2–5	3	5.6	0			0		3	6.0	
>5					0.008*					0.001*
**Metastatic lymph nodes**	15	27.8	20	55.6		23	57.5	12	24.0	
Negative	39	72.2	16	44.4		17	42.5	38	76.0	
Positive										

*P < 0.05.

**Table 2:** Correlative analysis of the miR-664b-5p expression with CCNE2 in TNBC tumour microarray.

	Tumour microarray (n = 90)		
	CCNE2 (Positive)		CCNE2 (Negative)
miR-664b-5p (high)	13		27
miR-664b-5p (low)	41		9
r		−0.502	
*P*		<0.001	
