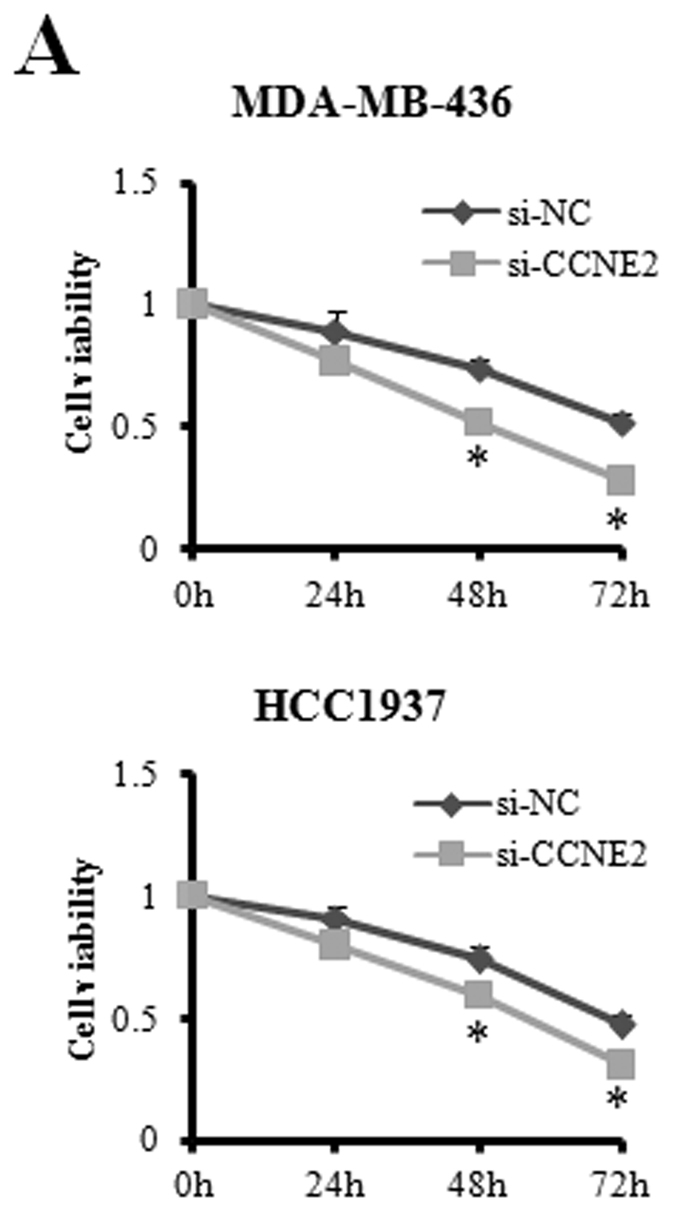# Corrigendum: PARP inhibitor increases chemosensitivity by upregulating miR-664b-5p in BRCA1-mutated triple-negative breast cancer

**DOI:** 10.1038/srep44346

**Published:** 2017-03-16

**Authors:** Wei Song, Lin Tang, Yumei Xu, Jing Xu, Wenwen Zhang, Hui Xie, Shui Wang, Xiaoxiang Guan

Scientific Reports 7: Article number: 4231910.1038/srep42319; published online: 02 08 2017; updated: 03 16 2017

This Article contains an error in Figure 4A where the graphs were labelled incorrectly. The correct Figure 4A appears below as Figure 1.

## Figures and Tables

**Figure 1:**