# Supplementary material for: PARP inhibitor increases chemosensitivity by upregulating miR-664b-5p in BRCA1-mutated triple-negative breast cancer
Source: Sci Rep. 2017 Feb 8;7:42319. doi: 10.1038/srep42319 (PMC5296748; doi:10.1038/srep42319)
Supplement: Supplementary Figures [file srep42319-s1.pdf]

PARP inhibitor increases chemosensitivity by upregulating miR-664b-5p in BRCA1-mutated triple-negative breast cancer

Wei Song[1], Lin Tang[2], Yumei Xu[1], Jing Xu[3], Wenwen Zhang[2], Hui Xie[4], Shui Wang[4], Xiaoxiang Guan[1,2]

[1]Department of Medical Oncology, Jinling Hospital, Southern Medical University, Guangzhou, 510515, China.

[2]Department of Medical Oncology, Jinling Hospital, Medical School of Nanjing University, Nanjing, 210002, China.

[3]Department of Medical Oncology, Jinling Hospital, Nanjing Medical University, Nanjing, 210002, China.

[4]Department of Breast Surgery, The First Affiliated Hospital of Nanjing Medical University, 300 Guangzhou Road, Nanjing, 210029, China.

Correspondence and requests for materials should be addressed to X. G. (email: xguan@nju.edu.cn) or S. W. (email: ws0801@hotmail.com).

S1

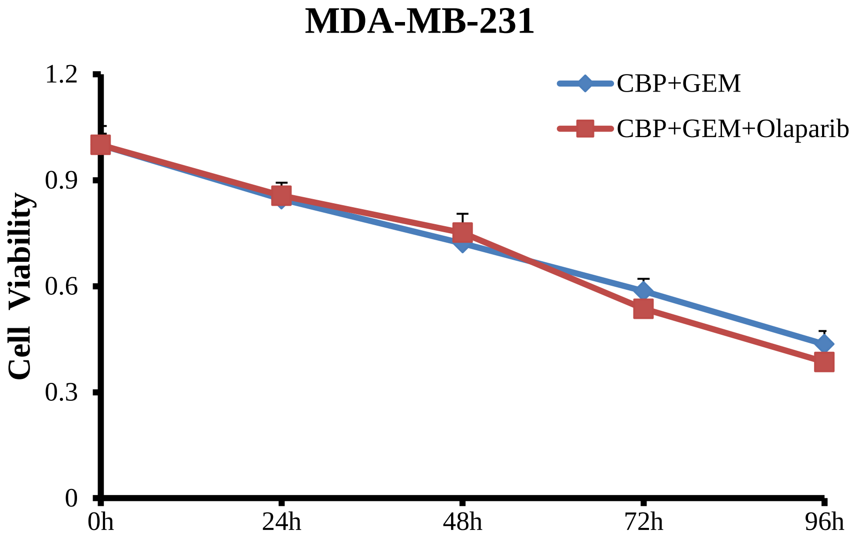

Supplementary Fig. 1. The non-BRCA1-mutated TNBC cell line MDA-MB-231 were treated with CBP (10 μM) plus GEM (100 nM) combined with or without olaparib (10 μM) for the indicated times, and then, the cell viability was determined with an MTT assay. The results show data from six independent experiments expressed as the mean ± SD. $p<0.05$.

S2

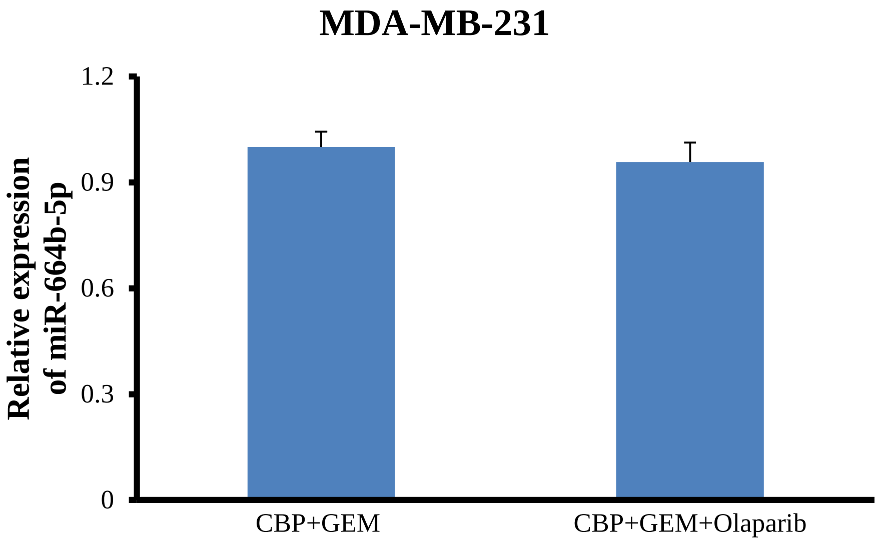

Supplementary Fig. 2. The expression of miR-664b-5p in non-BRCA1-mutated TNBC cell line MDA-MB-231 after treated with CBP plus GEM combined with or without olaparib was measured with qRT-PCR.
